# Semiautomated 3D Spine Reconstruction from Biplanar Radiographic Images: Prediction of Intervertebral Loading in Scoliotic Subjects

**DOI:** 10.3389/fbioe.2017.00001

**Published:** 2017-01-20

**Authors:** Tito Bassani, Claudia Ottardi, Francesco Costa, Marco Brayda-Bruno, Hans-Joachim Wilke, Fabio Galbusera

**Affiliations:** ^1^IRCCS Istituto Ortopedico Galeazzi, Milan, Italy; ^2^Laboratory of Biological Structure Mechanics, Department of Chemistry, Materials and Chemical Engineering ‘Giulio Natta’, Politecnico di Milano, Milan, Italy; ^3^Department of Neurosurgery, Humanitas Clinical and Research Center, Rozzano, Italy; ^4^Institute of Orthopaedic Research and Biomechanics, Centre for Trauma Research Ulm (ZTF), Ulm University, Ulm, Germany

**Keywords:** spine biomechanics, 3D model reconstruction, scoliosis, musculoskeletal modeling, spine loading prediction

## Abstract

The present study proposes a semiautomatic software approach to reconstruct 3D subject-specific musculoskeletal model of thoracolumbar spine from radiographic digitized images acquired with EOS system. The approach is applied to evaluate the intervertebral loads in 38 standing adolescents with mild idiopathic scoliosis. For each vertebra, a set of landmarks was manually identified on radiographic images. The landmark coordinates were processed to calculate the following vertebral geometrical properties in the 3D space (i) location (ii) dimensions; and (iii) rotations. Spherical joints simulated disks, ligaments, and facet joints. Body weight distribution, muscles forces, and insertion points were placed according to physiological–anatomical values. Inverse static analysis, calculating joints’ reactions in maintaining assigned spine configuration, was performed with AnyBody software. Reaction forces were computed to quantify intervertebral loads, and correlation with the patient anatomical parameters was then checked. Preliminary validation was performed comparing the model outcomes with that obtained from other authors in previous modeling works and from *in vivo* measurements. The comparison with previous modeling works and *in vivo* studies partially fulfilled the preliminary validation purpose. However, minor incongruities were pointed out that need further investigations. The subjects’ intervertebral loads were found significantly correlated with the anatomical parameters in the sagittal and axial planes. Despite preliminary encouraging results that support model suitability, future investigations to consolidate the proposed approach are necessary. Nonetheless, the present method appears to be a promising tool that once fully validated could allow the subject-specific non-invasive evaluation of a deformed spine, providing supplementary information to the routine clinical examination and surgical intervention planning.

## Introduction

Computational musculoskeletal modeling offers an invaluable insight to better understand spine loads in specific postures and pathological conditions (Adams and Dolan, 2005; Jalalian et al., 2013). Previous modeling works evaluated the thoracic region focusing on corrective treatments of scoliosis (Grealou et al., 2002; Aubin et al., 2003; Perie et al., 2003; Duke et al., 2005; Salmingo et al., 2012; Curtin and Lowery, 2014). Furthermore, spine models developed for load estimation were mostly oriented to explore lumbar region, describing thorax as a single rigid body (Stokes and Gardner-Morse, 1995; Shirazi-Adl et al., 2005; de Zee et al., 2007; Arjmand et al., 2009; Christophy et al., 2012; Han et al., 2012; Ghezelbash et al., 2016). Therefore, 3D subject-specific description of the whole thoracolumbar spine characterizing location and orientation of every vertebral level appears to be lacking in the literature. Such modeling approach would provide detailed investigation of the spinal loads acting in a deformed spine, e.g., in scoliosis, highlighting the correlations between anatomical parameters and loading alterations with respect to physiological condition. To this regard, adolescent idiopathic scoliosis represents a structural 3D deformity of the spine that in case of severe alterations may require surgical intervention to correct spine alignment (Weinstein et al., 2008; Nnadi and Fairbank, 2010; Gummerson and Millner, 2011). Monitoring scoliosis, the radiographic examination in coronal and sagittal planes is essential since it allows quantifying the anatomical properties, i.e., spine curves and vertebral rotations. Moreover, several non-invasive techniques based on computer vision have been proposed to monitor the postural asymmetries related to scoliosis (Aroeira et al., 2016). Unfortunately, both the radiographic analysis and the postural exams do not provide information about biomechanical aspects, i.e., loads acting on vertebrae and intervertebral disks, which may be correlated to the risk of curve progression. *In vivo* studies have been carried out to estimate internal loads indirectly by measuring intradiscal pressure (Schultz et al., 1982; Wilke et al., 2001; Polga et al., 2004; Meir et al., 2007), but the invasiveness of those methods make them inapplicable to adolescent scoliosis context. Conversely, 3D musculoskeletal modeling represents a non-invasive approach able to potentially provide essential subject-specific information on spinal loads, supporting clinicians in planning the best strategy for spinal instrumentation in case of scoliosis correction.

The present study proposes a semiautomatic software approach to reconstruct the 3D musculoskeletal model of thoracolumbar spine and pelvis from radiographic digitized images simultaneously acquired in coronal and sagittal planes. Subject-specific model reconstruction is performed evaluating 38 adolescent subjects suffering from mild scoliosis [Cobb angles (CAs) <24°] in standing position. Model suitability is performed assessing the correlation between intersegmental loads and anatomical parameters in the coronal, sagittal, and axial planes, calculated by evaluating the radiographic images. Model preliminary validation is performed comparing loads and muscle forces of a subject with CAs <5° with those predicted in physiological condition by El-Rich et al. (2004). Furthermore, the intervertebral disk pressure inferred by the proposed model is compared with the *in vivo* measurements performed in lumbar segment by Wilke et al. (2001) and by Sato et al. (1999), and in thoracic region by Polga et al. (2004).

## Methods

### Images Acquisition and Anatomical Parameters Extraction

Thirty-eight adolescent subjects [mean age 14 (SD 2); 27 females and 11 males] suffering from mild idiopathic scoliosis (CAs <24°) underwent radiographic examination in orthostatic position at IRCCS Istituto Ortopedico Galeazzi (Milan, Italy). The subject assent and the parental permission to use the anonymized radiological data were given by signing an informed consent approved by the local ethical committee. Digitized images of the thoracolumbar spine and pelvis were simultaneously acquired in coronal and sagittal planes with EOS imaging system (EOS Imaging, France). Since EOS system provides spatially calibrated images (Illés and Somoskeöy, 2012), no further calibration procedures were required. The images pair was manually processed through SterEOS software (EOS Imaging, France), which allowed to identify as “scoliotic,” the spine curves characterized by CAs >5°, and provided in addition the identification of the anatomical parameters. The following indexes were extracted for each subject: (i) number of scoliotic curves with related CA (in case that more than one curve had been identified, the most severe curve and related CA were chosen for the analyses); (ii) sacral slope (SS); (iii) pelvic incidence (PI); (iv) lumbar lordosis (LL); (v) thoracic kyphosis (TK) from T1 to T12; and (vi) maximum vertebral rotation in the axial plane (MAR). In addition, the Roussouly Type (RT) for the classification of the lumbar and pelvis sagittal alignment was manually determined (Roussouly et al., 2005).

### Geometric 3D Reconstruction

**Figure 1:**
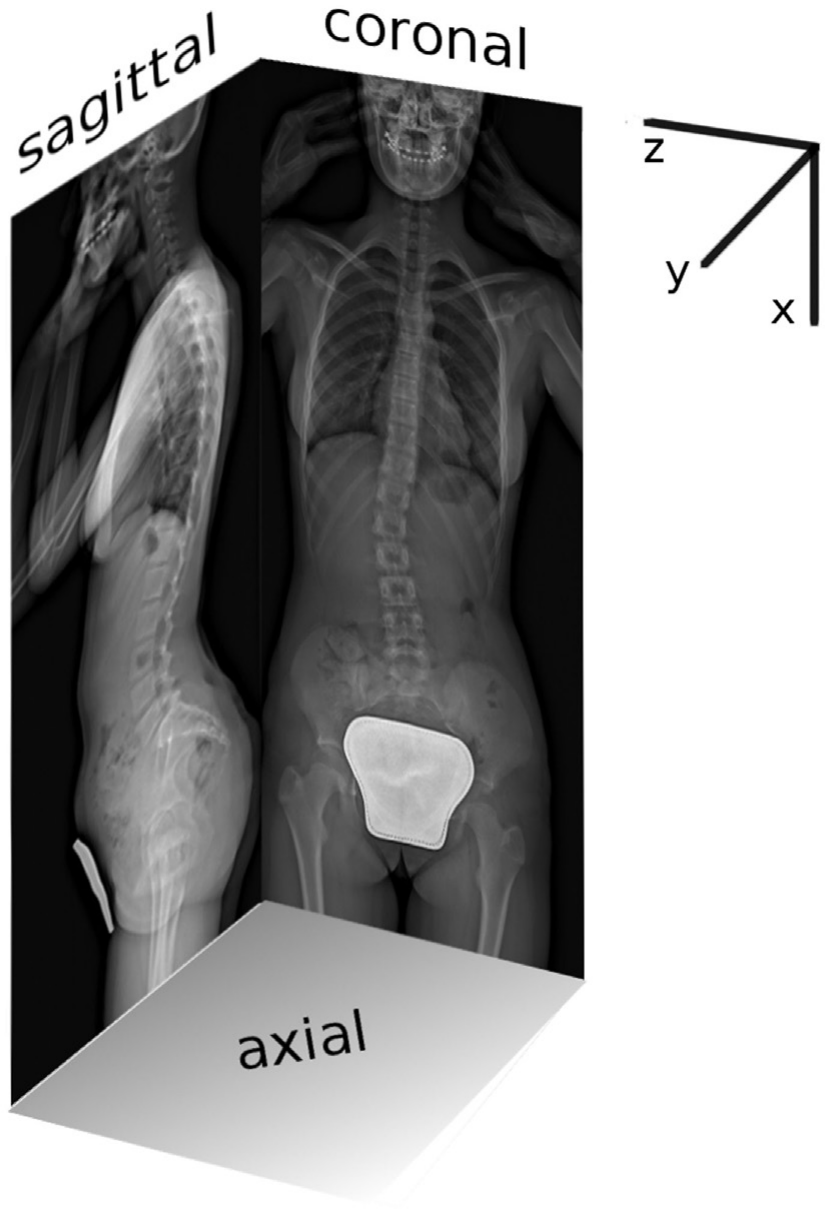
**Coronal and sagittal planar radiographic images acquired with EOS system as simultaneous projection of the global reference 3D system {*x,y,z*}**.

**Figure 2:**
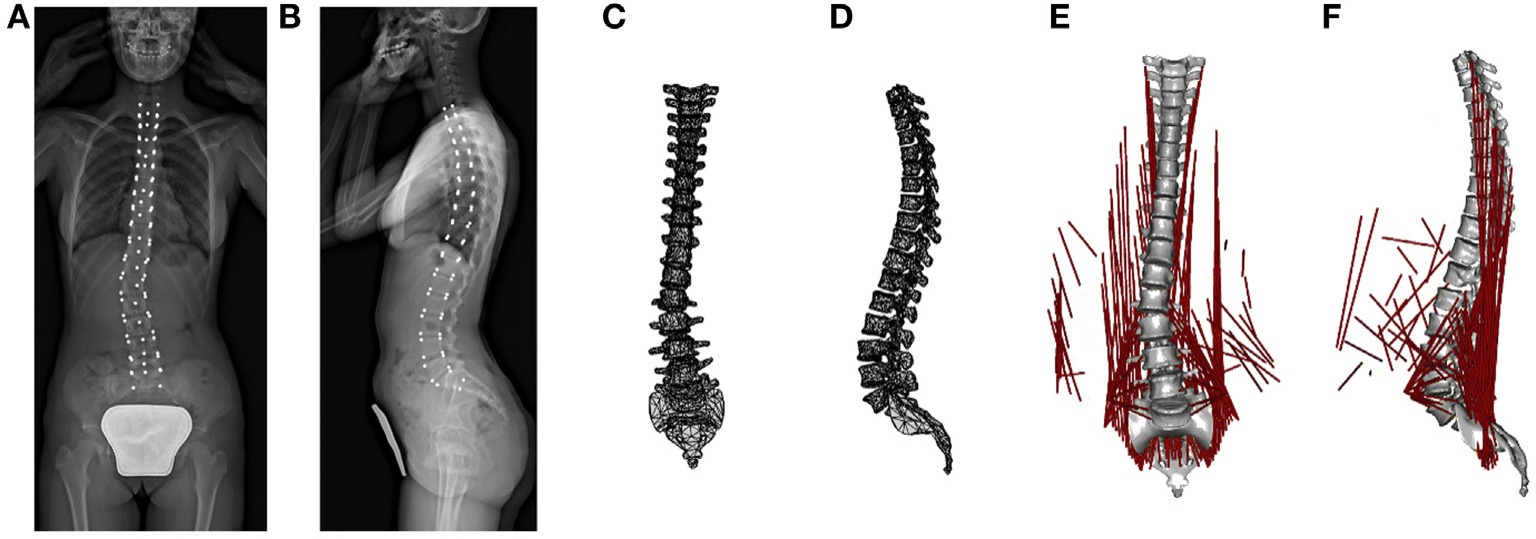
**From left to right: workflow summary of the model reconstruction**. The radiographic images with vertebral landmarks depicted in white **(A,B)**; the reconstructed 3D geometric model **(C,D)**; and the mechanical 3D model with muscles colored in red **(E,F)**.

The coronal and sagittal pair of biplanar images was manually processed with in-house script running with MATLAB (MathWorks Inc., Natick, MA, USA). Right-hand global reference system {*x,y,z*} was adopted considering axis “*x*” craniocaudally oriented, axis “*y*” posteroanteriorly, and axis “*z*” from right to left facing coronal plane (Figure 1). Since EOS system allows for simultaneous acquisition of true to size images in one-to-one scale, each point of the 3D space results jointly projected on the coronal and sagittal images (Figure 1). Accordingly, the identification of appropriate landmarks over the biplanar images allows for the calculation of geometrical parameters of spinal configuration of each vertebra in the 3D space using (i) location; (ii) dimensions; and (iii) rotation around the three axes. The Section “Appendix” explains in detail the reconstruction procedure. The 12 thoracic vertebrae (T1, …, T12), the 5 lumbar (L1, …, L5), and the first sacral (S1) were assessed (Figures 2A,B) obtaining the corresponding 3D geometric model (Figures 2C,D). It is worth noting that although visualized through a standardized set of reference anatomical vertebral meshes, the geometrical subject-specificity of the model is guaranteed by reconstructing vertebral location, dimensions, and rotations. The accuracy of the spine reconstruction method was previously checked by comparing the computed vertebral orientations with those obtained by SterEOS proprietary software, known suitable in providing accurate reconstruction of the spine (Glaser et al., 2012). The differences in reconstructing the orientation angles were found comparable with those presented by other authors when evaluating different reconstruction techniques (Pomero et al., 2004; Kadoury et al., 2009; Moura et al., 2011). Specifically, the difference in the thoracic section was 1.9° ± 0.4° (mean ± SD) for coronal rotation, 2.3° ± 0.8° for lateral rotation, and 3.7° ± 1.1° for axial rotation. Concerning the lumbar region, similar values were pointed out as 1.8° ± 0.6° for coronal rotation, 2.4° ± 0.9° for lateral rotation, and 3.6° ± 1.4° for axial rotation.

### Musculoskeletal Spine Model

Musculoskeletal modeling characterizes bones as rigid segments connected by joints and muscles as tensile elements attached to segments. Through an inverse dynamic approach, muscles forces and intersegmental forces acting during the execution of specific imposed kinematics and under the action of known external loads are computed by minimizing muscles recruitment activation (Rasmussen et al., 2001; Damsgaard et al., 2006). In the present work, the 3D musculoskeletal model was processed with AnyBody software v.6 (AnyBody Technology, Denmark). Geometric properties describing vertebral location, dimension, and rotations were set in AnyBody through MATLAB-based script, importing the geometric parameters as reconstructed in Section “Geometric 3D Reconstruction.” Accordingly, the spine mechanical model consisted of the thoracic and lumbar region, as well as the sacrum (Figures 2E,F). Sacrum was constrained to ground. Since the model was aimed to an inverse static simulation, vertebral mass and moments of inertia were set to 0. Conversely, mass of the body related to the trunk weight of the subject was distributed along the whole spine according to the literature (Kiefer et al., 1997). Weight distribution proposed by Kiefer et al. was scaled to match the subject weight thus improving the subject-specificity of the model. Weight forces were applied anteriorly to vertebrae, whereas weights related to head and arms were applied to T1 upper end-plate and anteriorly to T4, respectively (Kiefer et al., 1997). A total of 89 muscles and their attachment sites were identified according to physiologically–anatomically appropriate values (Stokes and Gardner-Morse, 1999) and incorporated into the spine model [12 muscle elements for thoracic multifidus (MF), 20 lumbar MF, 5 longissimus pars lumborum, 4 iliocostalis (IC) pars lumborum, 12 longissimus pars thoracis, 8 IC pars thoracis, 11 psoas, 5 quadratus lumborum, 6 external oblique, and 6 internal oblique]. Muscles were modeled as single force components exerting only tensile forces. No force–length relationship and no force–velocity relationship of muscles were modeled, since the present work evaluated exclusively static postures. Muscle wrapping around vertebral surfaces was not allowed by the current AnyBody release. Intervertebral disks were modeled as spherical joints with three rotational degrees of freedom, located at the midpoint of the segment connecting vertebral centers. Two adjacent vertebrae result thus connected by the joint through rigid subsegments starting in vertebral centers and ending in the joint. AnyBody force-dependent kinematic (FDK) analysis method minimizes muscles recruitment to calculate the intersegmental forces acting in maintaining the assigned configuration. In addition, the computed forces are iteratively processed to calculate joint translational deformation (i.e., shifts of the subsegments endings) *via* strain-displacement linear relation with imposed joint stiffness as weighting coefficient. As summarized in Table 1, joints’ translational stiffness interpreting soft tissues, i.e., disks and ligaments, and apophyseal joints’ role was set according to Panjabi et al. (1976) describing thoracic segments and according to Gardner-Morse and Stokes (2004) evaluating lumbar segments in preload condition (i.e., 500 N axial compressive) to describe orthostatic position. Rotational joint stiffnesses were not set since not accountable for by the current AnyBody release. The spherical joints provide thus internal reaction forces but not reaction moments. No tendons or other passive element properties were modeled. Muscle recruitment was set in order to minimize muscle activation *via* a polynomial optimization criterion, according to optimal delays in the muscle fatigue while maximizing muscle synergism (Rasmussen et al., 2001). It is worth noting that in the proposed modeling approach, the subject-specificity was accounted from geometrical and weight-distrubution points of view. Indeed, the vertebral location, orientation, and dimension were reconstructed from the radiographic images, and the subject weight was appropriately distributed along spine.

**Table 1:** **Translational joints stiffness related to axial (*x*′), coronal (*y*′), and sagittal (*z*′) local reference planes axes**.

	Translational stiffness (kN/m)		
	*x*′	*y*′	*z*′
Thoracic	943	86	101
Lumbar L1 to L3	2,420	397	523
Lumbar L4 to S1	2,420	473	523

*Values obtained evaluating adult subjects from Panjabi et al. (1976) describing thoracic joints and from Gardner-Morse and Stokes (2004) in preload condition (500 N axial compressive) describing lumbar joints*.

### Model Outputs

For each joint, the intersegmental force *F*, acting on the caudal vertebra was evaluated and measured in global reference system (*F_x_, F_y_, F_z_*), representing *F_x_* axial compression, *F_y_* posteroanterior shear, and *F_z_* lateral shear. Before being compared with other relevant variables, the resulting *F* values were normalized by the corresponding subject weight force in order to avoid potential biases related to the differences in the intersubjects’ weight. Possible correlations between *F* computed at L4L5 (*F*^L4L5^) and at L5S1 (*F*^L5S1^) with SS, PI, LL, and RT were searched. The *F* of spinal level corresponding to the scoliotic curve apex (*F*^A^) and the maximum of each component of *F* (*F*^M^) found along the scoliotic curve were checked for correlations with CA, TK, and MAR. Pearson correlation coefficient or Spearman rank correlation coefficient in case that a normal distribution was not achieved was accounted for in the comparisons. Both coefficients range from −1 to 1, where 0 indicates null linear correlation and −1 and 1 indicate full negative and positive linear correlations, respectively. Statistical significance of the coefficients was tested according to two-tailed *t*-test or permutation distribution testing assessing Pearson and Spearman coefficients, respectively, considering 0.05 as significance level.

### Model Preliminary Validation

Being {*x*′,*y*′,*z*′} the local vertebral coordinates system, the *F_x′_* and *F_y′_* acting on T12 and on lumbar vertebrae from L1 to L5, and the muscle forces calculated with the proposed model for a subject with no scoliotic curves (CAs <5°) were compared with the corresponding values computed by El-Rich et al. (2004) with a symmetric spine model representing healthy subject. The compared subject (female, 16 years, 154 cm of height, 47 kg of weight) was chosen as the most appropriate non-scoliotic subject suitable to guarantee comparable total weight distributed force in the two models (i.e., 345 and 387 N, respectively). The height of the spine model (from T1 to the sacrum) in the evaluated subject and in the model processed by El-Rich et al. resulted 39 and 47 cm, respectively. Unlike El-Rich et al. approach, in the present model the spine symmetry was not guaranteed, and muscle forces to be compared were obtained as the average of the left and right specific muscles values. According to the setting of El-Rich et al., the comparison was performed evaluating two different conditions: (i) gravity alone and (ii) adding to T3 a 380 N gravity-oriented load, accurately placed according to El-Rich et al., simulating a weighted bar held in front with arms extended in gravity direction close to the body. It is worth considering that there is a substantial difference between the proposed approach and that of El-Rich et al. in modeling intervertebral disks since the former defines them as deformable spherical joints, while the latter as flexible beams. According to that, the comparison of disks’ reaction moments provided by El-Rich et al. was not feasible since differently from the flexible beams the spherical joints are not able to provide reaction moments.

When comparing disk pressure computed by the present model with *in vivo* measurements, for every subject the pressure at the specific level was calculated as the ratio between the intersegmental axial load, *F_x′_*, acting on the caudal vertebra and the upper end-plate area of the caudal vertebra inferred from the geometrical reconstruction (see Appendix). Since recognized from *in vitro* studies that the pressure measured in the nucleus results higher than the average disk pressure (force divided by total cross-sectional area) by a factor of 1.54 (Brinckmann and Grootenboer, 1991; Nachemson, 1960), the computed pressure was accordingly corrected before being compared. Pressure at L4L5 was compared with *in vivo* measurements performed by Wilke et al. (2001) and by Sato et al. (1999). Thoracic pressure at T6T7, T7T8, T9T10, and T10T11 was compared with *in vivo* measurements obtained by Polga et al. (2004).

## Results

Comparing the proposed model with that of El-Rich et al. as preliminary validation, during both gravity alone and 380 N load conditions, *F_x′_* and *F_y′_* values were found comparable although larger values of *F_x′_* were found at L1 level and not increased progressively toward L5 as found in the El-Rich et al. model (Table 2). The muscle forces exhibited by the present approach at the different vertebral levels can be summarized as follows: (i) at T12 and L1, lower values during both conditions; (ii) at L2 and L3, comparable values in IC muscle and lower values in the other muscles during gravity alone, and larger values during 380 N load; and (iii) at L4 and L5, comparable values during gravity alone and larger values in 380 N load, with the exception of MF muscle at L5, which exhibited larger values during both conditions.

**Table 2:** **Intersegmental joint loads in local coordinates {*x*′,*y*′,*z*′} and muscle forces calculated for a subject with no scoliotic curves in comparison with corresponding values, depicted in italic, obtained by El-Rich et al. (2004)**.

	Gravity alone							380 N held in front						
	$F_{x^{\prime }}$		$F_{y^{\prime }}$		Muscle force			$F_{x^{\prime }}$		$F_{y^{\prime }}$		Muscle force		
T12	448	*336*	−70	−*38*	1	*14*	IC	1,773	*1,486*	−214	−*81*	126.7	*235*	IC
					<1	*34*	LG					4.7	*588*	LG
L1	557	*403*	−95	−*51*	<1	*7*	LG	2,001	*1,893*	−255	−*228*	<1	*51*	LG
					<1	*12*	MF					<1	*90*	MF
					<1	*8*	QL					<1	*60*	QL
L2	482	*445*	−83	−*71*	11	*9*	IC	1,700	*1,931*	−223	−*323*	36	*8*	IC
					<1	*4*	LG					15	*4*	LG
					1	*10*	MF					1	*9*	MF
					1	*3*	QL					18	*3*	QL
L3	450	*497*	−30	−*9*	11	*9*	IC	1,509	*1,954*	−86	*38*	27	*–*	IC
					1	*4*	LG					15	*–*	LG
					1	*14*	MF					2	*–*	MF
					3	*2*	QL					16	*–*	QL
L4	441	*534*	39	*31*	12	*7*	IC	1,383	*2,010*	88	*273*	35	*16*	IC
					8	*3*	LG					24	*7*	LG
					7	*9*	MF					17	*20*	MF
					7	*2*	QL					21	*4*	QL
L5	461	*575*	233	*218*	19	*28*	LG	1,326	*2,062*	551	*812*	41.3	*31*	LG
					110	*27*	MF					262.8	*55*	MF

*IC, iliocostalis; LG, longissimus; MF, multifidus; QL, quadratus lumborum; $F_{x^{\prime }}$, axial compression; $F_{y^{\prime }}$, posteroanterior shear*.

Comparing disk pressure calculated by the proposed model with that measured *in vivo* in lumbar and thoracic disks, pressure at L4L5 was found larger than that obtained by Wilke et al. and by Sato et al. (Table 3). In the thoracic region, when compared with that obtained by Polga et al., the model pointed out lower values in upper thoracic section (from T6 to T8) and comparable values in lower thoracic section (from T9 to T11).

**Table 3:** **Comparison of disk pressure calculated by the present model in lumbar and thoracic regions with that obtained from *in vivo* measurements by other authors evaluating relaxed standing position**.

	Disk	Pressure (MPa)	Disk section (cm^2^)	Subjects number	Age (years)	Weight (kg)
**Lumbar region**						
Present model	L4L5	0.78 (0.24)	11.8 (2.1)	37	14 (2)	47 (10)
Wilke et al. (2001)	L4L5	0.50	18.0	1	45	70
Sato et al. (1999)	L4L5	0.54 (0.18)	15.9 (1.8)	8	25 (22–29)	73 ± 11
**Thoracic region**						
Present model	T6T7	0.57 *(0.02)*	5.3 (1.0)	37	14 (2)	47 (10)
	T7T8	0.56 *(0.01)*	5.8 (1.1)			
	T9T10	0.83 *(0.03)*	6.9 (1.3)			
	T10T11	1.03 *(0.05)*	7.6 (1.6)			
Polga et al. (2004)	T6T7, T7T8^a^	1.01 *(0.06)*	–	6	28 (19–47)	73 (54–81)
	T9T10, T10T11^a^	0.86 *(0.06)*	–			

*Disk section areas, number of evaluated subjects, age, and weight are reported as well. Values are expressed as mean (SD) or mean (SE, in italic) where necessary for comparison*.

*^a^Pressure values calculated merging measurements from the two disks*.

**Figure 3:**
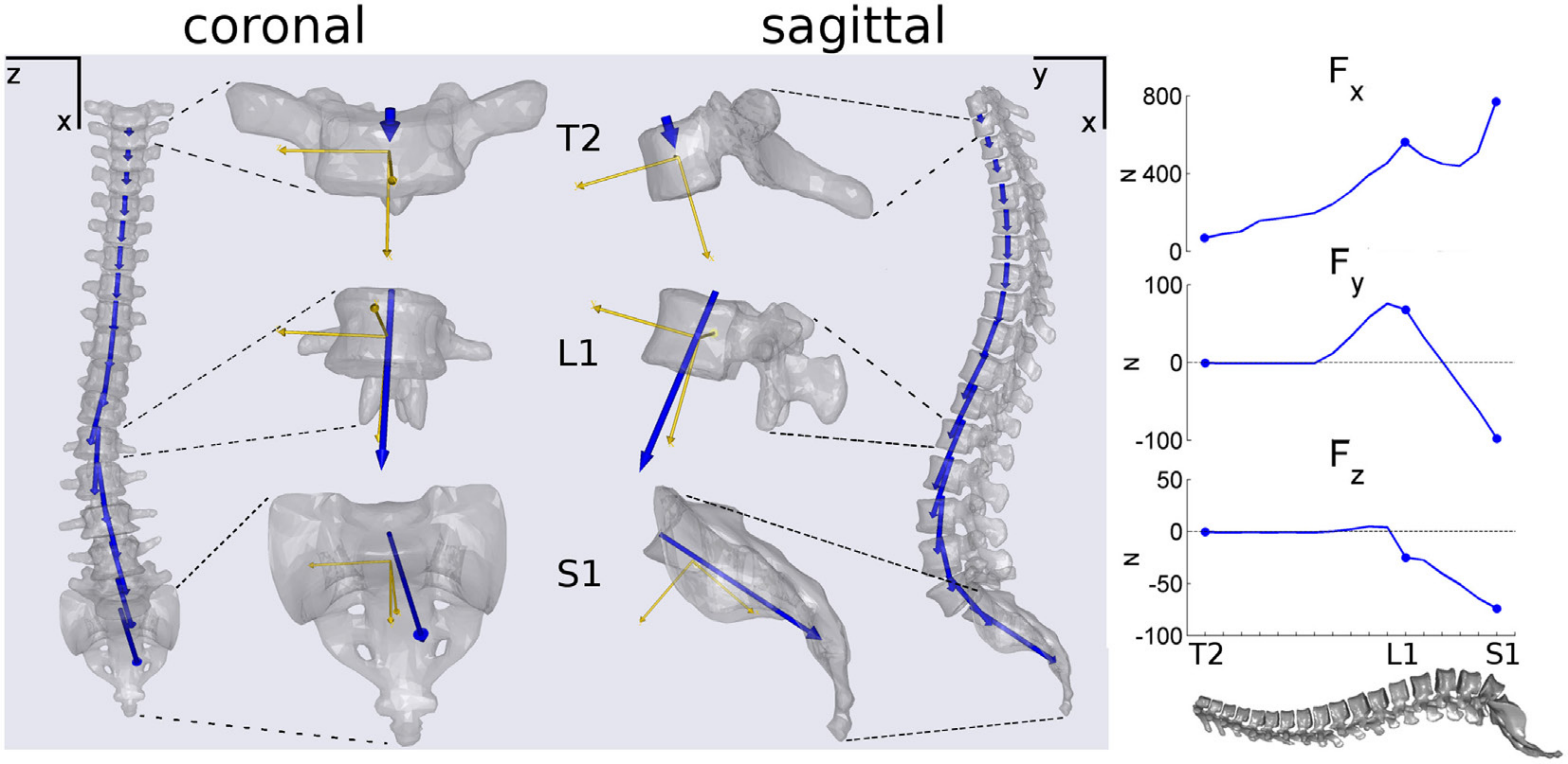
**Example illustrating coronal and sagittal projections of the spine and of disk joint load vectors *F* (in blue) calculated for a subject**. T2, L1, and S1 vertebrae are showed magnified depicting in addition the vertebral local reference systems (in yellow). In the right panel, progression of the *F* components in global coordinates {*x,y,z*} proceeding along spine from T2 to S1.

An example of the intersegmental joint force, *F*, computed for one subject is illustrated in Figure 3. As observable from coronal and sagittal projections, *F* vectors result substantially aligned with spine curvature. The axial load *F_x_* exhibits incremental values proceeding from T2 to S1 with a peak at T12–L1 level (right panel of Figure 3). Posteroanterior shear *F_y_* results positive in lower thoracic and upper lumbar segments and becomes negative in lower lumbar region. Lateral shear *F_z_* appears negligible in the thoracic spine and increases towards negative values proceeding along lumbar region.

**Figure 4:**
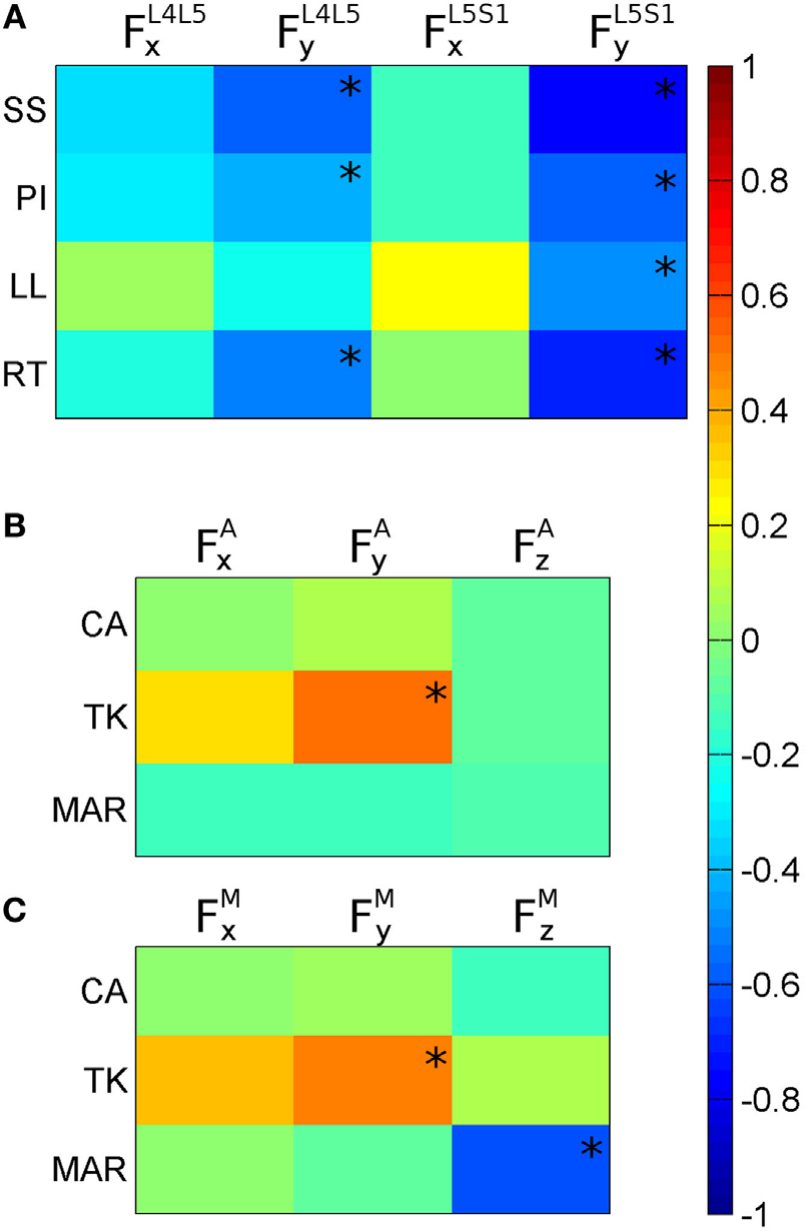
**Color maps of correlation coefficients calculated between anatomical parameters and joint internal loads (*F*)**. **(A)** Correlations of sacral slope (SS), pelvic incidence (PI), lumbar lordosis (LL), and Roussouly type (RT) with axial and posteroanterior loads in L4L5 $\left(F_{x}^{\text{L4L5}}\text{ and}F_{y}^{\text{L4L5}}\right)$ and in L5S1 $\left(F_{x}^{\text{L5S1}}\text{ and}F_{y}^{\text{L5S1}}\right)$. **(B)** Correlations of Cobb angle (CA), thoracic kyphosis (TK), and maximum axial rotation (MAR) with *F* components found in correspondence of the scoliotic curve apex (*F*^A^). **(C)** Correlations of CA, TK, and MAR with the maximum *F* components (*F*^M^) found inside the scoliotic curve. *indicates correlation statistical significance.

**Figure 5:**
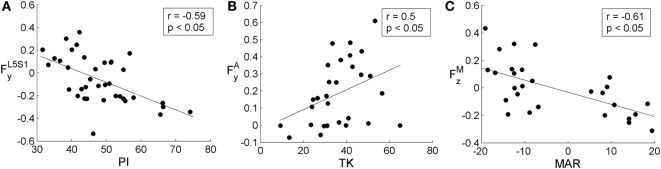
**Scatter plots showing in (A) the relation between pelvic incidence (PI) and posteroanterior load in L5S1 $\left(F_{y}^{L5S1}\right)$, in (B) between thoracic kyphosis (TK) and posteroanterior scoliotic curve apical load $\left(F_{y}^{A}\right)$, and in (C) between maximum axial rotation (MAR) and scoliotic curve maximum lateral load $\left(F_{z}^{M}\right)$**. All plots depict regression line and correlation coefficient, *r*, with the related statistical significance. PI, TK, and MAR are expressed in degrees. $F_{y}^{\text{L5S1}}$, $F_{y}^{\text{A}}$, and $F_{z}^{\text{M}}$ are expressed in adimensional units since divided by the corresponding subject weight force.

When assessing the entire group of subjects, correlation coefficients were calculated for 37 in 38 subjects (Figure 4A). One subject was excluded since corresponding mechanical model did not satisfy convergence criteria of AnyBody FDK approach. Results illustrated in Figures 4B,C accounted for subjects with at least one scoliotic curve identified (CA >5°). Accordingly, 29 in 38 subjects were assessed considering the subject unfulfilling FDK criteria once more excluded since no scoliotic curves could be detected. As shown in Figure 4A, significant correlations were found between anatomical parameters and L4L5 and L5S1 loads: (i) SS, PI, and RT with $F_{y}^{\text{L4L5}}$ and $F_{y}^{\text{L5S1}}$; (ii) LL with $F_{y}^{\text{L5S1}}$. As illustrated in Figures 4B,C, significant correlations were found with apical and maximum scoliotic curve loads, respectively, which are as follows: (i) TK with $F_{y}^{\text{A}}$ (Figure 4B) and $F_{y}^{\text{M}}$ (Figure 4C); (ii) MAR with $F_{z}^{\text{M}}$ (Figure 4C). Figure 5 describes the negative correlations found between PI and $F_{y}^{\text{L5S1}}$ (Figure 5A) and between MAR and $F_{z}^{\text{M}}$ (Figure 5C), and the positive correlation pointed out between TK and $F_{y}^{\text{A}}$ (Figure 5B). Since by definition *F_y_* exhibits positive values when posterioranteriorly oriented, Figures 5A,B show that the larger the PI and TK, the more $F_{y}^{\text{L5S1}}$ and $F_{y}^{\text{A}}$ resulted anteroposteriorly and posteroanteriorly oriented, respectively. Observing Figure 5C, it is worth noting that by definition MAR exhibits positive or negative values describing, respectively, right-hand and left-hand rotations around craniocaudal “*x*” axis. Negative slope of regression line between MAR and $F_{z}^{\text{M}}$ thus indicates incremental relation between MAR and the orientation of the maximum lateral intersegmental load $\left(F_{z}^{\text{M}}\right)$.

## Discussion

### Preliminary Model Validation

Intersegmental loads acting on T12 and in lumbar spine were compared with that computed by El-Rich et al. (2004) in two conditions: gravity alone and 380 N held in front load. As reported in Table 2, comparable values were pointed out assessing axial load, *F_x_*_′_, and posteroanterior shear, *F_y_*_′_. However, in the present model the maximum *F_x_*_′_ during the two conditions were found at L1 level instead progressively increasing from T12 to L5 as reported by El-Rich et al. This finding is confirmed evaluating the axial load in global coordinates (see *F_x_* in the lateral panel of Figure 3) describing the same subject in gravity alone condition. *F_x_* shows a peak at the thoracolumbar junction between T12 and L1, while larger value exceeding that peak was pointed out at the L5–S1 lumbosacral junction only (Figure 3). We hypothesize that this result can be related to the distribution of the muscles’ forces necessary to achieve equilibrium, which depending on the muscles configuration can potentially promote an increase of the axial load on T12–L1 junction. Further investigations are necessary to better clarify this outcome.

Muscle forces were found generally lower for gravity alone condition and mainly larger during 380 N load with respect to El-Rich et al. The larger values found in 380 N load can be explained considering that El-Rich et al. evaluated the model in an optimized posture aiming at minimizing loads, different from that assessed during gravity alone. Conversely, postures were kept identical in the present model. It is thus expected that a non-optimized model would exhibit higher muscle activations. Furthermore, perfect matching in the muscle comparison was not viable since muscles’ insertion placements were not identical in the compared models. In addition to muscles connecting the pelvis to thoracic and lumbar vertebrae, the present model accounted for intervertebral muscles (i.e., additional IC and longissimus pars thoracic, thoracic MF and lumbar MF muscles) that were not modeled by El-Rich et al., which can induce differences in the muscles forces distribution. Moreover, El-Rich et al. accounted for the entire T1–T12 segment as a rigid body, whereas the present approach assessed the thoracic vertebrae as separated bodies connected by spherical joints.

The calculated disk pressures were found comparable with that obtained *in vivo* in lower thoracic section (Table 3) thus supporting model suitability. However, pressures calculated in lumbar and upper thoracic sections were found 60% larger and 50% lower, respectively, in comparison with the *in vivo* measurements (Table 3). Although these discrepancies could be interpreted as effects of spine deformation characterizing scoliosis, further investigations are necessary to guarantee comprehensive model validation. For example, assessing the present approach in unload configuration, comparing pressures with the *in vivo* values measured by Meir et al. evaluating scoliotic patients in supine position during reconstructive surgery (Meir et al., 2007).

### Intersegmental Loads Orientation

The orientation of the intersegmental load, *F*, was found to be in principle aligned with spine curvature for every subject (Figure 3). This finding is in agreement with the follower load concept discussed in previous works (Patwardhan et al., 1999; Shirazi-Adl and Parnianpour, 2000) and supports the hypothesis that a load path tangent to the spine curve better sustains compression load (Patwardhan et al., 1999). However, this result can be mainly related to neglecting rotational joint stiffness because it is not allowed by AnyBody. Future developments are thus necessary to assess *F* orientation when adding intervertebral structures able to interpret passive rotational contributions (i.e., elastic elements).

For every subject, the incremental tendency of *F_x_* in the craniocaudal direction, related to the progressive increment of body weight to be sustained, exhibited a peak between T12 and L1 (right panel of Figure 3). Since body weight forces act along “*x*” axis and represent the solely external forces to be balanced in maintaining posture, the *F_x_* discontinuity can be related to the distribution of the muscles forces necessary to achieve equilibrium, which depending on the muscles’ configuration can potentially promote increased joint axial load on thoracolumbar junction. As stated in Section “Preliminary Model Validation” discussing model validation, further inquiries are necessary to better clarify this outcome.

### Correlating Lumbosacral Loads with Sagittal Parameters

The significant correlations found comparing SS, PI, and LL with the posteroanterior shear in L4L5 and L5S1 joints ($F_{y}^{\text{L4L5}}$ and $F_{y}^{\text{L5S1}}$, Figure 4A) support the suitability of the proposed model. The result is indeed in agreement with previous works (Galbusera et al., 2014) and suggests that the higher is the slope of sacrum and pelvis in sagittal plane, the more lumbosacral loads result anteroposteriorly oriented (i.e., showing negative values) in maintaining standing posture. That relation is verifiable observing the negative correlation between PI and $F_{y}^{\text{L5S1}}$ expanded in Figure 5A and can be elucidated through the example in Figure 3 where the *F* load acting on S1 in the sagittal projection appears anteroposteriorly oriented, and thus numerically negative. The correlation found between RT and $F_{y}^{\text{L4L5}}$ and $F_{y}^{\text{L5S1}}$ (Figure 4A) supports the relation identified between lumbosacral loads and anatomical sagittal parameters. By definition, Roussouly classification is indeed obtained evaluating SS together with lumbar curve characterization through the recognition of lumbar curve apex in sagittal plane (Roussouly et al., 2005) thus linking RT to both sacral and lumbar sagittal peculiarities.

### Correlating Loads in the Scoliotic Curve with Anatomical Parameters

The apical and the maximum loads inside the most severe scoliotic curve (*F*^A^ and *F*^M^) were compared with the anatomical parameters in the three anatomical planes (i.e., CA related to coronal plane, TK to sagittal, and MAR to axial). In coronal plane the CA index, which measures scoliosis severity, was found not correlated either with *F*^A^ or *F*^M^ (Figures 4B,C). This unexpected lack of correlation can be related to mild scoliosis condition, not able to induce significant increasing in lateral loads. Further developments evaluating severe scoliosis are thus required to verify this hypothesis. In sagittal plane, TK index was found correlated with apical and maximum posteroanterior shears ($F_{y}^{\text{A}}$ and $F_{y}^{\text{M}}$ in Figures 4B,C). In that case, as expanded in Figure 5B, the more TK was pronounced (i.e., larger TK values) the more $F_{y}^{\text{A}}$ resulted posteroanteriorly oriented. This finding confirms the relation between sagittal anatomical parameters and intersegmental posteroanterior shears, supporting the relation observed in Section “Correlating Lumbosacral Loads with Sagittal Parameters” between sagittal parameters and lumbosacral posteroanterior shears. In axial plane, the negative correlation found between MAR and maximum lateral shear $F_{z}^{\text{M}}$ (Figure 4C) is expanded in Figure 5C. This relation is explicable by observing the spine curvature depicted in the example of Figure 3. Although not scoliotic (CA <5°), this spine curve is characterized by positive axial rotations of L1 and L2 vertebrae (i.e., right-hand rotations around craniocaudal “*x*” axis) providing negative *F_z_* loads (right panel of Figure 3) oriented toward curve concavity. As inferable by Figure 5C, the larger was MAR the larger resulted *F_z_* oriented concavely to the curve.

Considering that a more severe scoliotic deformation is usually associated with larger vertebral axial rotations (Stokes et al., 1987), the significant correlations pointed out in the three anatomical planes corroborate previous reports about the importance of the 3D aspects of scoliotic deformity (Graf et al., 1983).

### Advantages and Limitations

The proposed modeling approach provides the following advances with respect to the state-of-the-art: (i) offers a non-invasive method to assess intersegmental loads, based on radiographic images evaluation; (ii) can be exploited to evaluate intersegmental loads in case of spine deformities, e.g., scoliosis, since characterizing each vertebral level of thoracolumbar spine; (iii) the reconstructed mechanical model is subject-specific from geometrical and weight-distrubution points of view, since vertebral geometrical location, orientation, and dimension are reconstructed evaluating the radiographic images, and subject weight is appropriately distributed along spine; (iv) the deformation of the intervertebral disks was accounted for through the AnyBody FDK method, which assesses force-dependent translational displacements by setting joint stiffnesses.

The present modeling approach has several limitations, which are as follows: (i) co-contraction of trunk muscles, known to be a physiological strategy to enhance spine stability (van Dieen et al., 2003), was neglected. Muscles were modeled as pure forces, not considering relevant aspects such as wrapping (Arjmand et al., 2006) and curved courses (Bazrgari and Shirazi-Adl, 2007); (ii) motion segments were modeled as kinematic joints with linear stiffness, thus neglecting real shape of the structure, the presence of posterior elements, and the non-linearity of biological soft tissues mechanics. However, the present model was designed to maintain standing posture thus avoiding the necessity of accounting for non-linear flexibility response of spinal segment (Abouhossein et al., 2011). The joint stiffness values chosen from the literature were measured in healthy young and middle-aged subjects without disk injuries or degenerative factors (Panjabi et al., 1976; Gardner-Morse and Stokes, 2004) since values from adolescents and scoliotic subjects are not available; (iii) rotational joint stiffness was neglected since not allowed by AnyBody. Further developments should include rotational parameters by purposely introducing intervertebral elastic elements; (iv) the description of spino-pelvic configuration was simplified to include only SS neglecting PI and hip axis; (v) the positions of muscle insertions were based on literature data and assumptions, which took into consideration only some aspects of patient-specific anatomy; (vi) rib cage modeling known affecting thoracic stiffness properties was neglected. Intra-abdominal pressure, recognized to play potential role on lumbar spine, was not accounted for since not expected to play significant role in relaxed standing.

In conclusion, the comparison with previous modeling works and *in vivo* studies partially fulfilled the preliminary validation purpose, but further investigations are necessary to clarify the observed incongruities and to guarantee comprehensive validation. When applying model approach to evaluate the relation between intersegmental loads and spine anatomical parameters in mild scoliotic subjects, encouraging results supporting model suitability were pointed out. Despite a number of limitations that suggest to be prudent and that will be overcome in future developments, the present method appears to be a promising tool. Once fully consolidated, it can allow the subject-specific non-invasive evaluation of a deformed spine, providing supplementary information to the routine clinical examination and supporting the surgical intervention planning.

## Ethics Statement

All images were taken from patients who signed an informed consent form, approved by the local ethical committee, which allowed subsequent use of their anonymized radiological data for retrospective studies.

## Author Contributions

TB wrote the manuscript; implemented the software procedures in MatLab and AnyBody; analyzed the data; and gave substantial contributions to the interpretation of the results. CO processed the radiographic images with the SterEOS software to obtain the anatomical parameters and performed the geometric reconstruction with the in-house MatLab script. FC drafted the manuscript and revised it critically for important intellectual content; gave substantial contribution to the interpretation of the results. MB-B, as spine surgeon, allowed for the acquisition of the radiographic images; supervised the clinical evaluation of the scoliosis parameters; and gave substantial contribution to the interpretation of the results. H-JW and FG analyzed the data; gave substantial contributions to the conception and design of the work and to the interpretation of the results. All the authors critically revised the manuscript and approved the version to be published.

## Conflict of Interest Statement

The authors declare that the research was conducted in the absence of any commercial or financial relationships that could be construed as a potential conflict of interest.

## Figures and Tables

**Figure A1:**
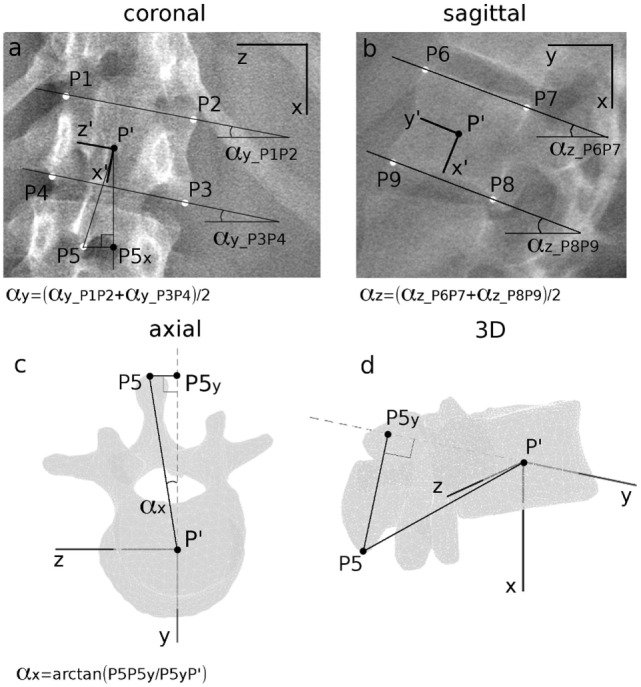
**Determination of coronal vertebral rotation α*_y_* (a) and of sagittal rotation α*_z_* (b) as the average of the slopes of the lines connecting upper and lower vertebral corners**. Determination of vertebral rotation in axial plane α*_x_* **(c)** obtained inferring the location of P5 in the axial plane **(c)** and in the 3D space **(d)** by evaluating geometric proprieties of a referential anatomic mesh model specific to the vertebra under assessment. Manually identified landmarks from P1 to P9 on coronal and sagittal images are depicted in white **(a,b)**, whereas calculated points are colored in black.

## Appendix

Figure A1 shows the approach performed for each vertebra to estimate the geometrical parameters. A set of nine landmarks (P1, …, P9) was manually identified obtaining the coordinates of points from P1 to P5 on the coronal image and from P6 to P9 on the sagittal image (Figures A1a,b). Vertebral location was estimated inferring the coordinates of vertebral center P′ as geometrical intersection between the vertebral body diagonals. Right-hand local reference system {*x*′,*y*′,*z*′} centered in P′ was defined to compute vertebral dimensions by evaluating the landmarks distances. Vertebral rotations in the three anatomical planes (α*_x_*, α*_y_*, α*_z_*) were calculated as described in the figure. Since an axial projection was not available, the estimation of vertebral rotation in the axial plane (α*_x_*) was based on a different approximate approach. For each specific vertebra, the corresponding reference anatomical 3D mesh model provided by SterEOS software was evaluated to predict the location of spinous process lower tip (P5) in the 3D space (Figures A1c,d). Those reference vertebral meshes were obtained through SterEOS software by reconstructing the spine of a 17-year-old adolescent male subject not affected by scoliosis. Specifically, those mesh models are created by SterEOS basing on a series of 3D CT models and a statistical finite element models (Illés and Somoskeöy, 2012) and are manually processed in the reconstruction procedure to match the vertebrae of the considered subject. The mesh dimensions were scaled to match the current vertebral body size. When projected on the axial plane, α*_x_* results computable as the arc tangent of the ratio between P5P5y and P5yP′ (Figure A1c) where both segments are estimable. P5P5y corresponds to P5P5x in the coronal projection (Figure A1a). P5yP′ corresponds to P5yP′ in the 3D space (Figure A1d) and can be inferable assessing the right-triangle P5-P5y-P′ where P5P′ is obtained measuring the scaled mesh model, and P5P5y corresponds to P5P′ in the coronal plane (Figure A1a). It is worth noting that the axial rotation (α*_x_*) was thus computed under the assumption that the coordinates of the spinous process of the reference mesh, after properly being scaled, matched the coordinates of the spinous process of the subject in the radiographic images. In this regard, the evaluated subjects were previously checked to not present vertebral deformities in order to satisfy the assumed anatomical matching condition. This requirement was verified by the orthopedic surgeon responsible of the clinical examinations.
